# Farmer-reported chicken-health challenges and perceived dust exposure near a mine tailings facility

**DOI:** 10.3389/fvets.2026.1956527

**Published:** 2026-10-22

**Authors:** Tshegofatso Shirus Machubeng, Zwelethu Mfanafuthi Mdletshe, Tlou Grace Manyelo, Busisiwe Gunya

**Affiliations:** Department of Agricultural Economics and Animal Production, Faculty of Science and Agriculture, University of Limpopo, Sovenga, South Africa

**Keywords:** chicken health, farmer perceptions, indigenous chickens, mine tailings facility, perceived dust exposure, smallholder production systems

## Abstract

Mine tailings facilities can generate airborne particulate matter with potential implications for environmental quality and livestock health. This study assessed farmer-perceived dust exposure, reported chicken-health challenges, and poultry-management practices among households located at different distances from a chromite mine tailings facility. A cross-sectional survey was conducted from May to August 2025 among 202 indigenous-chicken farmers in three spatial groups located approximately 1.96, 6.60, and 18.24 km from the facility, corresponding to 1–5 km (*n* = 70), 6–10 km (*n* = 77), and >10 km (*n* = 55), respectively. A 17-item semi-structured questionnaire assessed household and flock characteristics, management practices, perceived environmental dust conditions, farmer-reported chicken-health indicators, mortality, and treatment practices. Distance groups were compared using Kruskal–Wallis tests followed, where significant, by Holm-adjusted pairwise Wilcoxon rank-sum tests. Binary logistic regression evaluated factors associated with farmer-reported chicken-health challenges. Farmer-perceived dust accumulation, dust visibility, and environmental contamination differed among distance groups (all *p* < 0.0001), as did chicken-health challenges, nasal discharge, reduced activity, number of visibly affected birds, mortality, and use of traditional remedies (*p* ≤ 0.0092). Visibly affected birds decreased progressively across distance groups, whereas mortality and traditional-remedy use had higher mean ranks in the 1–5-km group. Conversely, perceived environmental indicators had their highest mean ranks in the >10-km group, indicating no simple distance-response pattern. In the multivariable model, perceived dust visibility (OR = 0.08, 95% CI: 0.03–0.19) and environmental contamination (OR = 0.14, 95% CI: 0.08–0.27) were negatively associated with reported chicken-health challenges, whereas visibly affected birds were positively associated (OR = 1.45, 95% CI: 1.15–1.82). Mortality and traditional-remedy use were also associated with the outcome, although the latter estimate was imprecise (OR = 20.8, 95% CI: 1.20–360). These cross-sectional, farmer-reported findings provide baseline observational evidence and do not establish causal or seasonal relationships. Structured farmer reporting may complement veterinary surveillance in smallholder poultry systems. Future studies should integrate seasonal surveys with environmental monitoring, clinical assessment, laboratory diagnostics, and spatial exposure analysis.

## 1 Introduction

Indigenous chickens are important to food security, household income, and livelihood resilience in smallholder farming communities, yet their health is frequently constrained by limited access to veterinary services, diagnostic facilities, biosecurity, and structured disease surveillance (1–3). Village-poultry studies in Ghana and Zimbabwe have documented the importance of free-range and scavenging production systems to rural households, while broader assessments highlight their contributions to food security, income generation, and small-scale poultry development (13, 16, 18, 21, 23). Under scavenging and semi-scavenging systems, birds spend substantial periods outdoors and may, therefore, encounter diverse environmental, infectious, nutritional, and management-related health risks. South African indigenous chickens also represent important genetic resources whose conservation and sustainable utilisation remain relevant to the resilience of rural poultry-production systems (19, 20).

Airborne particulate matter represents one potential environmental challenge to poultry health. The avian respiratory system is highly specialised for efficient gas exchange, but this anatomical arrangement may also increase susceptibility to inhaled particulate material (4, 5). Exposure to elevated particulate concentrations in poultry environments has been associated with respiratory irritation and respiratory-health disorders (6, 7). However, respiratory-health problems in poultry may involve multiple risk factors, as illustrated by epidemiological investigations of respiratory infections in poultry production systems (17). These concerns may be relevant to smallholder flocks located near mine tailings facilities, where exposed tailings surfaces can generate and disperse dust under dry and windy conditions (8–10, 22).

In resource-constrained livestock systems, farmers are often the first observers of changes in animal behaviour, clinical signs, mortality, and treatment needs. Farmer-derived information can, therefore, complement formal veterinary surveillance by identifying locally important health concerns and potential exposure patterns requiring further investigation (11, 12). However, farmer-reported signs are not equivalent to clinical diagnoses, and perceived environmental exposure does not establish the source or magnitude of exposure. Questionnaire-based findings should, therefore, be interpreted as epidemiological screening information rather than confirmation of disease causation.

Despite growing concerns regarding mining-associated environmental contamination, limited information is available on farmer-reported poultry-health challenges in smallholder communities surrounding mine tailings facilities. In particular, few studies have examined whether reported flock-health problems and perceived environmental dust exposures vary across households situated at different distances from such facilities.

Therefore, this study assessed farmer-reported chicken-health challenges, perceived environmental dust exposure, and selected poultry-management characteristics among smallholder households located at different distances from a chromite mine tailings facility in Limpopo Province, South Africa. We hypothesised that farmer-reported chicken-health challenges and perceived environmental dust exposure would differ among distance zones and that perceived dust-exposure indicators would be associated with the likelihood of reporting chicken-health challenges.

## 2 Materials and methods

### 2.1 Study area and poultry-production systems

The study was conducted in rural communities surrounding a chromite mine tailings facility in Limpopo Province, South Africa (24.55932° S, 30.15034° E). The three surveyed household groups were located approximately 1.96, 6.60, and 18.24 km from the mine tailings facility, respectively, corresponding to the analytical distance categories of 1–5, 6–10, and >10 km used throughout the study. Household surveys were conducted from May to August 2025, corresponding to the cool-dry period in the Southern Hemisphere. This period is characterised by limited rainfall, dry exposed surfaces, and commonly windy conditions, which may favour the mobilisation and dispersion of loose particulate material. Indigenous chickens were predominantly managed under extensive scavenging or semi-scavenging systems, with birds generally allowed to forage outdoors during the day and confined in household shelters at night. These terms describe the predominant management context reported by participating households, particularly daytime scavenging and nighttime confinement; the production-system category was not treated as a separate exposure classification in the statistical analyses. Figure 1 shows the mine tailings facility, surveyed communities, and distribution of participating households across the three distance categories.

**Figure 1:**
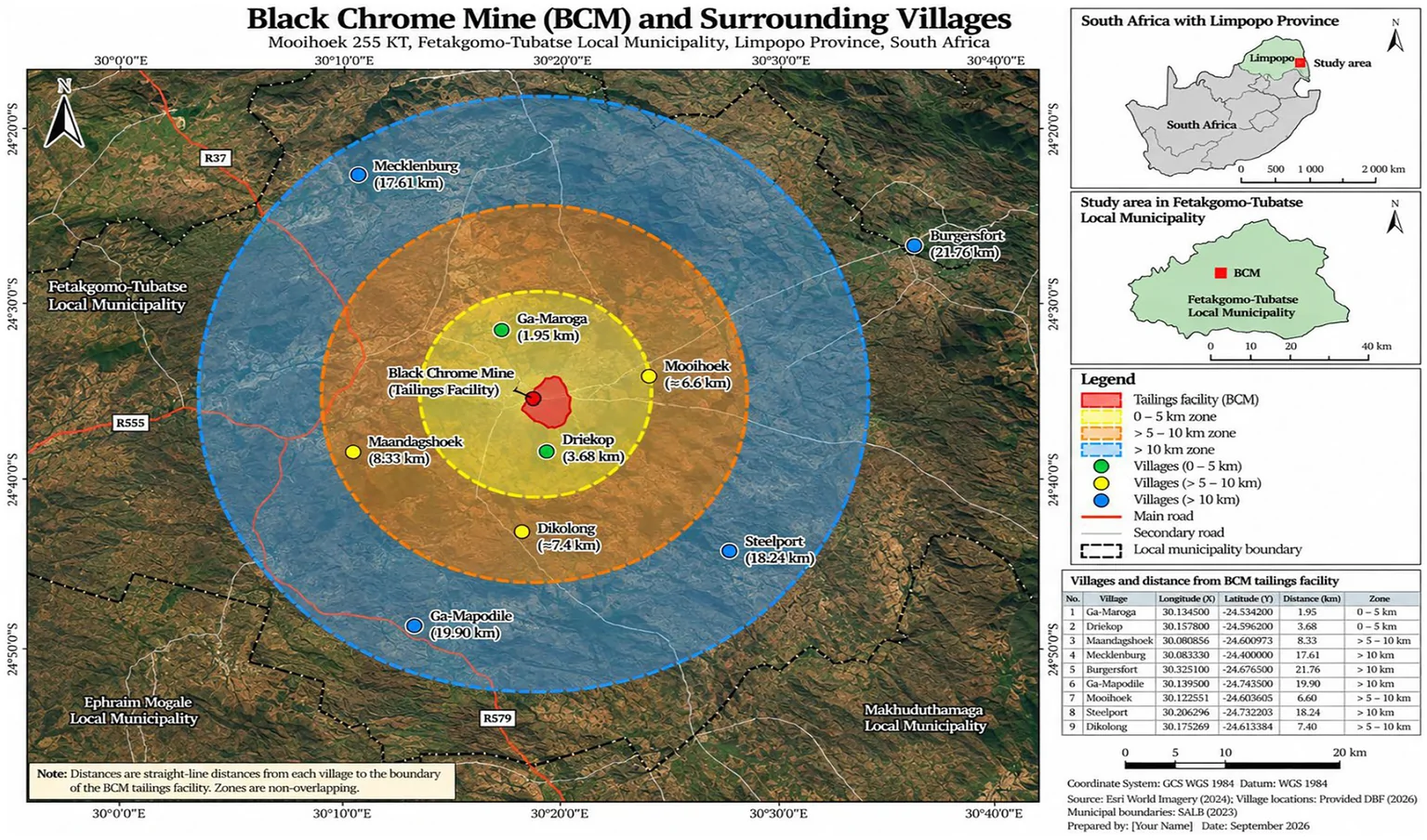
Figure shows the mine tailings facility, surveyed communities, and their spatial relationship to the three predefined distance categories.

### 2.2 Study design, household selection, and data collection

A cross-sectional household survey was conducted from May to August 2025 during the local cool-dry period. Local traditional leaders, agricultural extension officers, and community livestock groups assisted in identifying eligible indigenous-chicken-keeping households. Households were eligible if they were located within one of the predefined study areas, kept indigenous chickens at the time of the survey, and had an adult household member who was responsible for or knowledgeable about poultry management and willing to provide informed consent. Households were classified into three spatial groups according to their location relative to the mine tailings facility: 1–5 km (*n* = 70), 6–10 km (*n* = 77), and >10 km (*n* = 55). The surveyed household groups were located approximately 1.96, 6.60, and 18.2 km from the facility, respectively. These categories were used solely as spatial grouping variables and were not considered validated measures of particulate exposure. Because environmental particulate concentrations and composition, wind speed and direction, other meteorological conditions, and source-specific dust dispersion were not measured, increasing distance from the facility was not assumed to represent decreasing environmental dust exposure.

A 17-item semi-structured questionnaire was administered face-to-face to an adult household member responsible for, or knowledgeable about, poultry management. The questionnaire collected information on respondent and household characteristics, poultry-management practices, perceived environmental dust conditions, farmer-observed chicken-health signs, mortality, and treatment practices. Health-related variables included coughing, nasal discharge, swollen eyes, reduced activity, number of visibly affected birds, and farmer-reported mortality. Perceived environmental variables included dust accumulation, dust visibility, and environmental contamination. Only variables prospectively collected through the original 17-item questionnaire were included in the present analyses; no additional respondent or management variables were retrospectively reconstructed.

The study was designed as a farmer-report survey and did not include direct veterinary clinical examination, laboratory diagnosis, necropsy, or environmental particulate measurement. Accordingly, farmer-reported health signs represent farmer observations rather than confirmed disease diagnoses, while perceived dust exposure represents respondent-reported environmental conditions rather than measured particulate exposure. The complete 17-item questionnaire is provided as Supplementary Material S1. Questionnaire-derived variables included in the statistical analyses, together with their response options, analytical coding, and measurement structure, are summarised in Supplementary Table S1.

### 2.3 Ethical considerations

Ethical approval was obtained from the Turfloop Research Ethics Committee of the University of Limpopo (Protocol No. TREC/775/2025: UG). Participation was voluntary, and informed consent was obtained from all respondents before completion of the questionnaire. To ensure confidentiality, personal identifiers were excluded from the dataset, and all responses were anonymised before statistical analysis. Respondents were informed that participation was voluntary and that they could decline to answer any question or withdraw from the survey at any stage without consequence.

### 2.4 Statistical analysis

All questionnaire data were analysed using Statistical Analysis Software (SAS Institute Inc., Cary, NC, USA; SAS, 2025). Descriptive statistics were used to summarise household and flock characteristics, poultry-management practices, farmer-perceived environmental conditions, and farmer-reported chicken-health indicators. Because the questionnaire-derived variables were ordinal or did not satisfy assumptions of normality, differences among the three predefined distance groups (1–5, 6–10, and >10 km) were evaluated using the Kruskal–Wallis test. Where the overall Kruskal–Wallis test was significant, pairwise comparisons between distance groups were performed using Wilcoxon rank-sum tests, with *p*-values adjusted for multiple comparisons using the Holm method. Results are presented as mean ranks, Kruskal–Wallis *χ*^2^ statistics, and *p*-values. Holm-adjusted *p* < 0.05 was considered statistically significant for pairwise comparisons.

A binary logistic regression model (PROC LOGISTIC) was used to evaluate factors associated with farmer-reported chicken-health challenges. The dependent variable was whether a household reported chicken-health challenges (1 = yes; 0 = no). Candidate questionnaire-derived variables were screened using the preceding between-group analyses, and variables showing statistically significant differences were considered for the multivariable model. The final model included perceived dust visibility, perceived environmental contamination, number of visibly affected birds, mortality, and use of traditional remedies. No automated forward, backward, or stepwise variable-selection procedure was applied. Because the number of visibly affected birds, mortality, and use of traditional remedies were measured concurrently with the reported health outcome, these variables were included as associated questionnaire-derived indicators or management responses rather than as temporally antecedent causal predictors. Associations are presented as adjusted odds ratios (ORs) with corresponding 95% confidence intervals (CIs). Given the cross-sectional study design, model estimates were interpreted as associations and not as evidence of causal relationships.

The logistic regression model was expressed as:

$$\text{logit}(P_{i})=\ln[P_{i}/(1-P_{i})]=\beta_{0}+\beta ₁X{₁}_{i}+\beta ₂X{₂}_{i}+\ldots +\beta_{k}X_{\mathrm{ki}}.$$

where Pᵢ is the probability that household *i* reported chicken-health challenges; β₀ is the intercept; β₁ to β_k_ are the regression coefficients; and X₁ᵢ to X_ki_ represent the explanatory variables included in the model. Model estimates are presented as odds ratios (OR) with corresponding 95% confidence intervals (CI). Given the cross-sectional study design, model estimates were interpreted as associations and not as evidence of causal relationships.

## 3 Results

### 3.1 Household and flock characteristics

Household size (*χ*^2^ = 2.7323, *p* = 0.2551) and household income (*χ*^2^ = 0.1722, *p* = 0.9175) did not differ among distance groups (Table 1). Flock size differed among distance groups (*χ*^2^ = 17.1859, *p* = 0.0002). Pairwise comparisons showed that flock size differed between the 1–5- and 6–10-km groups (Holm-adjusted *p* < 0.0001), whereas neither the 1–5- versus >10-km comparison (*p* = 0.0908) nor the 6–10- versus >10-km comparison (*p* = 0.4975) was significant.

**Table 1:** Household and indigenous-chicken flock characteristics according to distance from the chromite mine tailings facility.

Variable	1–5 km (*n* = 70) mean rank	6–10 km (*n* = 77) mean rank	>10 km (*n* = 55) mean rank	*χ^2^*	*p*-value
Household size	110	99.8	93.1	2.73	0.2551
Household income	104	100	101	0.17	0.9175
Flock size	124	84.5	96.9	17.2	0.0002

Values are mean ranks from the Kruskal–Wallis test. Where the overall test was significant, pairwise comparisons among distance groups were performed using Wilcoxon rank-sum tests with Holm adjustment for multiple comparisons. Mean ranks within a row that do not share a common superscript letter differ significantly at Holm-adjusted *p* < 0.05. Household size refers to the number of people residing in the household. *χ*^2^, Kruskal–Wallis chi-square statistic.

### 3.2 Farmer-perceived environmental dust exposure

Farmer-perceived environmental dust exposure differed among distance groups (Table 2). Dust accumulation (*χ*^2^ = 32.2239, *p* < 0.0001), dust visibility (*χ*^2^ = 130.4698, *p* < 0.0001), and perceived environmental contamination (*χ*^2^ = 109.6234, *p* < 0.0001) differed among groups. For each indicator, all three pairwise comparisons remained significant after Holm adjustment (*p* ≤ 0.0034). Mean ranks increased across the distance groups, with the lowest ranks in the 1–5-km group and the highest in the >10-km group.

**Table 2:** Farmer-perceived environmental dust exposure according to distance from chromite mine tailings facility.

Variable	1–5 km (*n* = 70) mean rank	6–10 km (*n* = 77) mean rank	>10 km (*n* = 55) mean rank	*χ* ^2^	*p*-value
Dust accumulation	75.5^c^	105^b^	130^a^	32.2	< 0.0001
Dust visibility	51.5^c^	104^b^	162^a^	131	< 0.0001
Environmental contamination	53.8^c^	105^b^	158^a^	110	< 0.0001

Values are mean ranks from the Kruskal–Wallis test. Where the overall test was significant, pairwise comparisons among distance groups were performed using Wilcoxon rank-sum tests with Holm adjustment for multiple comparisons. Mean ranks within a row that do not share a common superscript letter differ significantly at Holm-adjusted *p* < 0.05. Variables represent farmer-perceived environmental conditions and should not be interpreted as direct measurements of particulate exposure. *χ*^2^, Kruskal–Wallis chi-square statistic.

### 3.3 Farmer-reported chicken-health indicators, mortality and treatment practices

Farmer-reported chicken-health challenges differed among distance groups (*χ*^2^ = 36.7835, *p* < 0.0001; Table 3). The 6–10-km group had a lower mean rank than both the 1–5- and >10-km groups (both Holm-adjusted *p* < 0.0001), whereas the latter two groups did not differ (*p* = 0.3891). Nasal discharge also differed overall (*χ*^2^ = 9.9348, *p* = 0.0070); however, only the 1–5- and >10-km groups differed after Holm adjustment (*p* = 0.0035).

**Table 3:** Farmer-reported chicken-health indicators, mortality, and treatment practices according to distance from the chromite mine tailings facility.

Indicator	1–5 km (*n* = 70) mean rank	6–10 km (*n* = 77) mean rank	>10 km (*n* = 55) mean rank	*χ* ^2^	*p*-value
Chicken-health challenges	114^a^	75.8^b^	122^a^	36.8	<0.0001
Nasal discharge	85.7^b^	105^ab^	117^a^	9.93	0.0070
Reduced activity	115^a^	102^ab^	83.4^b^	9.39	0.0092
Number of visibly affected birds	140^a^	96.2^b^	60.2^c^	60.5	<0.0001
Mortality	140^a^	86.7^b^	73.9^b^	55.1	<0.0001
Use of traditional remedies	116^a^	95.4^b^	91.5^b^	25.3	<0.0001

Values are mean ranks from the Kruskal–Wallis test. Where the overall test was significant, pairwise comparisons among distance groups were performed using Wilcoxon rank-sum tests with Holm adjustment for multiple comparisons. Mean ranks within a row that do not share a common superscript letter differ significantly at Holm-adjusted *p* < 0.05. Health-related variables represent farmer-reported observations and were not confirmed by veterinary clinical examination or laboratory diagnosis. *χ*^2^, Kruskal–Wallis chi-square statistic.

Reduced activity differed among distance groups (*χ*^2^ = 9.3850, *p* = 0.0092), with a significant difference between the 1–5- and >10-km groups (Holm-adjusted *p* = 0.0076); the 6–10-km group did not differ from either group. The number of visibly affected birds differed markedly among distance groups (*χ*^2^ = 60.4813, *p* < 0.0001), and all three pairwise comparisons remained significant after Holm adjustment (all *p* < 0.0001).

Mortality also differed among groups (*χ*^2^ = 55.0600, *p* < 0.0001). The 1–5-km group had a higher mean rank than both the 6–10- and >10-km groups (both Holm-adjusted *p* < 0.0001), whereas the latter two groups did not differ (*p* = 0.1994). Use of traditional remedies differed among groups (*χ*^2^ = 25.2676, *p* < 0.0001); the 1–5-km group differed from both the 6–10-km (*p* = 0.0007) and >10-km (*p* = 0.0003) groups, whereas the latter two groups did not differ (*p* = 0.1426).

### 3.4 Factors associated with farmer-reported chicken-health challenges

Multivariable binary logistic regression identified significant associations between farmer-reported chicken-health challenges and perceived dust visibility, perceived environmental contamination, number of visibly affected birds, mortality, and use of traditional remedies (Table 4). The estimate for traditional-remedy use had a wide confidence interval (95% CI = 1.20–360), indicating substantial uncertainty in the magnitude of this association.

**Table 4:** Multivariable logistic regression analysis of factors associated with farmer-reported chicken-health challenges in indigenous-chicken flocks.

Predictor	Adjusted odds ratio	95% CI	*p*-value
Perceived dust visibility	0.08	0.03–0.19	<0.0001
Perceived environmental contamination	0.14	0.08–0.27	<0.0001
Number of visibly affected birds	1.45	1.15–1.82	0.0015
Mortality (0 vs. 1–5 birds)	0.33	0.12–0.90	0.0310
Use of traditional remedies	20.8	1.20–360	0.0368

OR, odds ratio; CI, confidence interval. Adjusted ORs and 95% CIs were estimated using multivariable binary logistic regression. OR > 1 indicates higher odds and OR < 1 indicates lower odds of reporting chicken-health challenges relative to the specified reference category or per-unit increase in a continuous variable, as applicable. Exact Wald p-values are reported. Given the cross-sectional design, these estimates represent associations and should not be interpreted as causal relationships. Statistical significance was set at *p* < 0.05.

## 4 Discussion

The present study identified distinct spatial patterns in farmer-perceived environmental conditions and farmer-reported chicken-health indicators among households located at different distances from the chromite mine tailings facility. Perceived dust accumulation, dust visibility, and environmental contamination differed across all three distance groups, with mean ranks increasing from the 1–5-km to the >10-km group. In contrast, the number of visibly affected birds decreased progressively across distance groups, while mortality was higher by mean rank in the 1–5-km group than in the two more distant groups. Farmer-reported chicken-health challenges followed a non-linear spatial pattern: the 6–10-km group had a lower mean rank than both the 1–5- and >10-km groups, which did not differ from each other. These contrasting patterns indicate that geographical distance from the tailings facility did not correspond to a uniform gradient across perceived environmental conditions and reported flock-health indicators.

Farmer-reported nasal discharge was ranked higher among households located >10 km from the facility, whereas reduced activity, numbers of visibly affected birds, mortality, and use of traditional remedies were ranked higher among households within 1–5 km. Respiratory signs such as nasal discharge may occur in response to airborne irritants but are clinically non-specific and may also be associated with infectious disease, housing conditions, nutritional status, or other environmental exposures. Although particulate matter has been associated with poultry respiratory-health impairment (6, 7), the present study did not include clinical examination or laboratory diagnosis. The reported signs should, therefore, be interpreted as farmer-observed flock-health indicators rather than evidence of a specific respiratory disease or mine-tailings-related pathology.

All three farmer-perceived environmental indicators differed across each pair of distance groups. Mean ranks for dust accumulation, dust visibility, and environmental contamination were lowest in the 1–5-km group, intermediate in the 6–10-km group, and highest in the >10-km group. This pattern does not support the assumption that households geographically closest to the tailings facility necessarily experienced the greatest perceived environmental dust conditions. Because particulate concentrations, wind speed and direction, and source-specific dust dispersion were not measured, the observed spatial pattern cannot be attributed directly to emissions from the tailings facility. Other environmental factors, including prevailing winds, topography, vegetation cover, land use, and additional local dust sources, may have contributed to the reported conditions; however, these remain hypotheses requiring direct investigation.

The logistic-regression findings further demonstrate the complexity of relationships among questionnaire-derived environmental perceptions, flock-health observations, and management responses. The negative associations of perceived dust visibility and environmental contamination with reported chicken-health challenges do not support a simple interpretation in which greater perceived dust corresponds to greater reported flock-health problems. Conversely, the positive association with the number of visibly affected birds is consistent with households being more likely to recognise and report a flock-health challenge when a greater number of birds show observable signs. Mortality and use of traditional remedies should similarly be interpreted in the context of recognition and management of flock-health problems rather than as independent causal determinants. Because these variables were measured concurrently, their temporal ordering cannot be established.

Use of traditional remedies was associated with farmer-reported chicken-health challenges. In smallholder poultry systems, farmer experience, locally available remedies, access to veterinary services, and perceived disease severity often shape treatment decisions. Experience with Newcastle disease control in village chickens further highlights the importance of practical, sustainable disease-management approaches adapted to local production conditions (14). The present association should, therefore, be interpreted as a marker of treatment-seeking behaviour rather than evidence that traditional remedies caused or prevented the disease. Moreover, the wide confidence interval surrounding this estimate indicates substantial uncertainty in the magnitude of the association and warrants cautious interpretation.

Farmer-reported flock-health indicators did not show a single spatial pattern. The number of visibly affected birds differed among all three distance groups and decreased progressively with increasing distance. Mortality was higher by mean rank in the 1–5-km group than in both more distant groups, whereas the 6–10- and >10-km groups did not differ. Reduced activity differed only between the 1–5- and >10-km groups, while the intermediate group did not differ from either. Farmer-reported chicken-health challenges showed a different pattern, with the 6–10-km group having a lower mean rank than both the nearest and most distant groups. These findings indicate that the observed flock-health patterns cannot be explained by geographical distance alone and may reflect multiple environmental, husbandry, infectious, nutritional, and management-related influences.

The importance of maintaining indigenous-chicken health also extends 319 beyond flock survival and household livelihoods, as South African research has documented distinct consumer sensory characteristics of indigenous chicken meat compared with conventional broiler meat (15). Nevertheless, meat quality and consumer preferences were not assessed in the present study. Several limitations should be considered when interpreting these findings. Environmental particulate concentrations and composition were not measured; therefore, the predefined distance categories represent geographical groupings rather than measured exposure levels. Although exposure to certain heavy metals can produce toxicological effects (24), this study did not determine the elemental composition or bioavailability of airborne dust, nor did it measure metal exposure in chickens. Farmer-reported chicken-health indicators were not independently confirmed through clinical examination, laboratory diagnosis, or post-mortem investigation. The cross-sectional design also precludes determination of temporal or causal relationships among perceived environmental conditions, flock-health indicators, mortality, and treatment practices. The questionnaire also relied on respondents’ retrospective reporting of respiratory illness and mortality during the preceding 12 months, potentially introducing recall and reporting bias. Furthermore, ocular and nasal discharge were recorded as a combined questionnaire response, preventing separate evaluation of these clinical signs. Differences between original questionnaire coding and analytical recoding must be considered when interpreting ordinal environmental indicators and regression estimates.

Furthermore, meteorological conditions, topography, vegetation, and other potential environmental dust sources were not quantified. The findings should therefore be interpreted as farmer-derived observational evidence that generates hypotheses for subsequent environmental and veterinary investigation.

Future investigations should integrate farmer-based surveillance with clinical flock examination, diagnostic testing, necropsy where appropriate, environmental particulate monitoring, meteorological measurements, and repeated seasonal sampling. Such an integrated veterinary epidemiological approach would help distinguish environmental, infectious, and management-related determinants of poultry-health challenges and provide stronger evidence for targeted flock-health interventions in mining-affected rural communities.

## 5 Conclusion

This cross-sectional study identified spatial differences in farmer-perceived environmental conditions and farmer-reported chicken-health indicators among indigenous-chicken-keeping households located at different distances from a chromite mine tailings facility. Farmer-perceived dust accumulation, dust visibility, and environmental contamination differed across all three distance groups and had their highest mean ranks in the >10-km group. In contrast, the number of visibly affected birds decreased progressively across distance groups, while mortality and use of traditional remedies were higher by mean rank in the 1–5-km group than in the two more distant groups. Farmer-reported chicken-health challenges followed a non-linear spatial pattern, with the 6–10-km group differing from both the 1–5- and >10-km groups. Collectively, these contrasting patterns do not demonstrate a simple distance-response relationship between proximity to the tailings facility, perceived environmental dust conditions, and reported chicken-health challenges. The findings provide farmer-derived observational evidence that can inform future studies integrating direct particulate measurements, meteorological data, veterinary clinical assessment, and longitudinal monitoring to clarify potential environmental exposure pathways and their relationships with poultry health.

## Data Availability

The original contributions presented in the study are included in the article/Supplementary material, further inquiries can be directed to the corresponding author.

## References

[1] Alders RG Pym RAE. Village poultry: still important to millions, eight thousand years after domestication. Worlds Poult Sci J. (2009) 65:181–90. doi: 10.1017/S0043933909000117

[2] Kpomasse CC Kouame YAE N’nanle O Houndonougbo FM Tona K Oke OE. The productivity and resilience of the indigenous chickens in the tropical environments: improvement and future perspectives. J Appl Anim Res. (2023) 51:456–69. doi: 10.1080/09712119.2023.2228374

[3] Padhi MK. Importance of indigenous breeds of chicken for rural economy and their improvements for higher production performance. Scientifica. (2016) 2016. doi: 10.1155/2016/2604685, 27144053 PMC4838803

[4] Fedde MR. Relationship of structure and function of the avian respiratory system to disease susceptibility. Poultr Sci. (1998) 77:1130–8. doi: 10.1093/ps/77.8.1130, 9706077

[5] Maina JN. Perspectives on the structure and function of the avian respiratory system: functional efficiency built on structural complexity. Front Anim Sci. (2022) 3:851574. doi: 10.3389/fanim.2022.851574

[6] Almuhanna EA Ahmed AS Al-Yousif YM. Effect of air contaminants on poultry immunological and production performance. Int J Poult Sci. (2011) 10:461–70. doi: 10.3923/ijps.2011.461.470

[7] Wang K Shen D Dai P Li C. Particulate matter in poultry house on poultry respiratory disease: a systematic review. Poult Sci. (2023) 102:102556. doi: 10.1016/j.psj.2023.102556, 36848758 PMC9982681

[8] Csavina J Field J Félix O Corral-Avitia AY Sáez AE Betterton EA. Effect of wind speed and relative humidity on atmospheric dust concentrations in semi-arid climates. Sci Total Environ. (2012) 433:58–73. doi: 10.1016/j.scitotenv.2014.03.138, 24769193 PMC4072227

[9] Del Rio-Salas R Ayala-Ramírez Y Loredo-Portales R Romero F Molina-Freaner F Minjarez-Osorio C . Mineralogy and geochemistry of rural road dust and nearby mine tailings: a case of ignored pollution hazard from an abandoned mining site in semi-arid zone. Nat Resour Res. (2019) 28:1485–503. doi: 10.1007/s11053-019-09472-x

[10] Mpanza M Adam E Moolla R. Perceptions of external costs of dust fallout from gold mine tailings: west Wits Basin. Clean Air J. (2020) 30:1–12. doi: 10.17159/caj/2020/30/1.7566

[11] Catley A Alders RG Wood JLN. Participatory epidemiology: approaches, methods, experiences. Vet J. (2012) 191:151–60. doi: 10.1016/j.tvjl.2011.03.010, 21856195

[12] Mariner JC Paskin RD. Manual on Participatory Epidemiology: Methods for the Collection of action-Oriented Epidemiological Intelligence. Rome: Food and Agriculture Organization (2000).

[13] Aboe PAT Boa-Amponsem K Okantah SA Butler EA Dorward PT Bryant MJ. Free-range village chickens on the Accra Plains, Ghana: their husbandry and productivity. Trop Anim Health Prod. (2006) 38:235–48. doi: 10.1007/s11250-006-4356-x, 16986771

[14] Alders RG Bagnol B Young MP. Technically sound and sustainable Newcastle disease control in village chickens: lessons learnt over fifteen years. Worlds Poult Sci J. (2018) 74:433–40. doi: 10.1017/S0043933918000439

[15] Dyubele NL Muchenje V Nkukwana TT Chimonyo M. Consumer sensory characteristics of broiler and indigenous chicken meat: a south African example. Food Qual Prefer. (2010) 21:815–9. doi: 10.1016/j.foodqual.2010.04.005

[16] Guèye EF. The role of family poultry in poverty alleviation, food security and the promotion of gender equality in rural Africa. Outlook Agric. (2000) 29:129–36. doi: 10.5367/000000000101293130

[17] Kadja MC Anitcheou J Djossa GJ Sabi SS Laleye FX Kane Y . Risk factors associated with respiratory infections in poultry in peri-urban areas of Dakar and Thies (Senegal). Asian J Med Health. (2021) 19:69–77. doi: 10.9734/ajmah/2021/v19i730345

[18] Melesse A. Significance of scavenging chicken production in the rural community of Africa for enhanced food security. Food Sci Qual Manag. (2014) 24:41–7. doi: 10.1017/S0043933914000646

[19] Mogano RR Mpofu TJ Mtileni B Hadebe K. South African indigenous chickens' genetic diversity, and the adoption of ecological niche modelling and landscape genomics as strategic conservation techniques. Poult Sci. (2025) 104:104508. doi: 10.1016/j.psj.2024.104508, 39657468 PMC11681890

[20] Mtileni BJ Muchadeyi FC Maiwashe A Chimonyo M Dzama K. Conservation and utilisation of indigenous chicken genetic resources in South Africa. Worlds Poult Sci J. (2010) 66:415–28. doi: 10.1017/S0043933912000852

[21] Muchadeyi FC Sibanda S Kusina NT Kusina J Makuza S. The village chicken production system in Rushinga District of Zimbabwe. Livest Res Rural Dev. (2004) 16:40. Available online at: https://www.lrrd.cipav.org.co/lrrd16/6/much16040.htm

[22] Oguntoke O Ojelede ME Annegarn HJ. Frequency of mine dust episodes and the influence of meteorological parameters on the Witwatersrand area, South Africa. Int J Atmos Sci. (2013) 2013:128463. doi: 10.1155/2013/128463

[23] Sonaiya EB Swan SEJ. Small-scale Poultry Production: Technical guide, vol. 1 Rome, Italy: Food and Agriculture Organization (2004).

[24] Tchounwou PB Yedjou CG Patlolla AK Sutton DJ. Heavy metal toxicity and the environment. EXS. (2012) 101:133–64. doi: 10.1007/978-3-7643-8340-4_6, 22945569 PMC4144270

