# Supplementary material for: Farmer-reported chicken-health challenges and perceived dust exposure near a mine tailings facility
Source: Front Vet Sci. 2026 Oct 22;13:1956527. doi: 10.3389/fvets.2026.1956527 (PMC13735576; doi:10.3389/fvets.2026.1956527)
Supplement: Supplementary file 1 [file Supplementary_file_1.docx]

**Questionnaire**

**Section A: Demographic and Production Profile**

1. Village/Location: ___________________
2. Household size: ______
3. Primary income source:
   1. ☐ Crop farming 2. ☐ Livestock 3. ☐ Mining
4. Number of chickens owned: _______
5. Production system:
   1. ☐ Extensive (free-ranging)	2. ☐ Semi-intensive	3. ☐ Intensive

**Section B: Environmental Exposure to Tailing Dust**

6. Estimated distance from the nearest tailing facility:
   1. ☐ <1 km	2. ☐ 1–5 km	3. ☐ 6–10 km	4. ☐ >10 km
7. Frequency of dust accumulation on household surfaces:
   1. ☐ Daily	2. ☐ Weekly	3. ☐ Occasionally	4. ☐ Never
8. Visibility of airborne dust during dry seasons:
   1. ☐ Highly visible	2. ☐ Moderately visible	3. ☐ Rarely visible	4. ☐ Not visible
9. Perception of environmental contamination caused by mining dust:
   1. ☐ Severe	2. ☐ Moderate	3. ☐ Mild	4. ☐ None

**Section C: Poultry Health and Respiratory Indicators**

10. Has respiratory illness been observed in your flock in the past 12 months?
    1. ☐ Yes	2. ☐ No
11. Common symptoms observed (tick all that apply):
    1. ☐ Coughing	2. ☐ Sneezing	3. ☐ Ocular/nasal discharge	4. ☐ Laboured breathing	5. ☐ Decreased activity
12. Number of birds affected in the last episode: ______
13. Number of mortality cases attributed to respiratory illness in past 12 months:
    1. ☐ 0	2. ☐ 1–5	3. ☐ 6–10	4. ☐ >10
14. Interventions used:

1. ☐ Antimicrobials	2. ☐ Traditional remedies	3. ☐ None	4. ☐ Both

**Section D: Perceptions and Environmental Correlations**

15. Have you observed an increase in respiratory illness during periods of high dust activity?
    1. ☐ Yes	2. ☐ No	3. ☐ Not sure
16. Do you believe tailing dust contributes to the health problems in your flock?
    1. ☐ Yes	2. ☐ No	3. ☐ Uncertain
17. Would you be willing to allow environmental or biological sampling at your premises for further analysis?
    1. ☐ Yes	2. ☐ No
