# Supplementary material for: Farmer-reported chicken-health challenges and perceived dust exposure near a mine tailings facility
Source: Front Vet Sci. 2026 Oct 22;13:1956527. doi: 10.3389/fvets.2026.1956527 (PMC13735576; doi:10.3389/fvets.2026.1956527)
Supplement: Supplementary file 2 [file Table_1.docx]

Supplementary Table S1. Questionnaire-derived variables, original response options, analytical coding, and measurement structure used to assess farmer-perceived environmental conditions and reported indigenous-chicken health indicators near a chromite mine tailings facility.

| Questionnaire item | Variable | Original response options and codes | Analytical coding or verification required | Measurement structure |
| --- | --- | --- | --- | --- |
| Q1 | Village/location | Open-ended location | Spatial group assignment to be verified | Nominal |
| Q2 | Household size | Number of household members | Recorded count | Discrete count |
| Q3 | Primary income source | 1 = Crop farming; 2 = Livestock; 3 =Mining | Categorical; verify Table 1 variable | Nominal |
| Q4 | Flock size | Number of chickens owned | Recorded count | Discrete count |
| Q5 | Production system | 1 = Extensive; 2 = Semi-intensive; 3 = Intensive | Original categories | Ordinal/categorical |
| Q6 | Estimated distance | 1 = <1 km; 2 = 1–5 km; 3 = 6–10 km; 4 = >10 km | Final three groups: 1–5, 6–10, >10 km; verify exclusion/reclassification of <1 km | Ordinal spatial category |
| Q7 | Dust accumulation | 1 = Daily; 2 = Weekly; 3 = Occasionally; 4 = Never | Verify whether original or reverse coding was analysed | Ordinal |
| Q8 | Dust visibility | 1 = Highly visible; 2 = Moderately visible; 3 = Rarely visible; 4 = Not visible | Verify original/reverse coding and logistic-regression contrast | Ordinal |
| Q9 | Perceived environmental contamination | 1 = Severe; 2 = Moderate; 3 = Mild; 4 = None | Verify original/reverse coding and logistic-regression contrast | Ordinal |
| Q10 | Reported respiratory illness | 1 = Yes; 2 = No | Manuscript outcome: 1 = Yes; 0 = No, if recoded from Q10 | Binary |
| Q11 | Reported clinical signs | Coughing; sneezing; ocular/nasal discharge; laboured breathing; decreased activity; multiple selections permitted | Individual indicators require binary coding; verify ocular/nasal discharge label | Multiple-response nominal |
| Q12 | Visibly affected birds | Number affected during last episode | Recorded count; confirm model scale | Discrete count |
| Q13 | Reported mortality | 1 = 0; 2 = 1–5; 3 = 6–10; 4 = >10 birds | Verify categories, reference group and contrasts in Table 4 | Ordinal |
| Q14 | Interventions used | 1 = Antimicrobials; 2 = Traditional remedies; 3 = None; 4 = Both | Traditional-remedy use: verify whether categories 2 and 4 were combined | Nominal; potentially derived binary |
| Q15 | Perceived illness during high-dust periods | 1 = Yes; 2 = No; 3 = Not sure | Not included in reported main analyses | Nominal |
| Q16 | Belief that tailings dust affects flock health | 1 = Yes; 2 = No; 3 = Uncertain | Not included in reported main analyses | Nominal |
| Q17 | Willingness to permit sampling | Yes; No | Not included in reported main analyses | Binary |

Original response categories correspond to the 17-item questionnaire provided in Supplementary Material S1. Multiple-response clinical signs were recorded under questionnaire item 11. The distance categories represent spatial groupings rather than measured particulate exposure. Questionnaire response codes are distinguished from analytical coding where transformations were applied. Variables not included in the reported analyses are identified accordingly.
